# UV-C treatment promotes quality of early ripening apple fruit by regulating malate metabolizing genes during postharvest storage

**DOI:** 10.1371/journal.pone.0215472

**Published:** 2019-04-16

**Authors:** Jakaria Chowdhury Onik, Yajing Xie, Yuquan Duan, Xiaojia Hu, Zhidong Wang, Qiong Lin

**Affiliations:** Institute of Food Science and Technology, Chinese Academy of Agricultural Sciences/Key Laboratory of Agro-products Quality and Safety Control in Storage and Transport Process, Ministry of Agriculture and Rural Affairs, Beijing, China; Beijing Forestry University, CHINA

## Abstract

Early ripening apples are usually used for fresh marketing because of short storage life, although they are with high acid and low sugar contents. Understanding the malate metabolism in fleshy fruit and underpinning process during ripening is crucial for particular crop improvement where acidity is a concern for direct consumption or further processing. In this research, a traditional Chinese apple cultivar ‘Hongyu’, which belongs to early ripening apple cultivar, were freshly harvested at commercial maturity stage (120 Days after full bloom) and used for different storage temperature (4°C, 20°C) and UV-C treatment (following storage at 20°C after treatment). Simple sugars (glucose, sucrose, and fructose) and organic acids (malic, and oxalic) were assessed after 14 d of storage. Compared to fruits stored at 20°C, the malate content in fruits stored at 4°C significantly higher, while it was decreased significantly in UV-C treated fruits stored at 20°C after 14 d of storage. The sugar content was almost similar throughout the UV-C-treated fruits and fruits stored at different temperature. The higher ratios of total sugars to total organic acids in UV-C treated fruits after 14 d suggest that UV-C treatment has the potential to improve the taste of early ripening apple cultivars. Considering the significant difference in malate the samples at 14 d of storage were subjected for RNA-seq analysis. Transcriptome analysis revealed that the phenomena underlying this change were governed by metabolism of malate by the regulation of NADP-malic enzyme (*NADP-ME*) and phosphoenolpyruvate carboxylase kinase (*PEPCK*) in apple during postharvest storage. This transcriptome profiling results have specified the transcript regulation of malate metabolism and lead to possible taste improvement without affecting the other fruit quality attributes.

## Introduction

Apple (*Malus domestica* Borkh.) is an economically important and popularly grown fruit crop all over the world due to high nutritive value, good taste and availability throughout the year. The postharvest storage period varied among different cultivars and different harvesting time [1]. In general, early harvested apple cultivars such as ‘Gala’, ‘Royal Gala’ tended to be relatively less suitable for the long-term cold storage, comparing the late harvested apple cultivars such as ‘Fuji’, ‘Red Delicious’, ‘Golden Delicious’ [2,3,4]. Therefore, summer apple cultivars or early harvested apple cultivars have been in sale for fresh market but not for long-term storage [1]. ‘Granny Smith’ apples, which were belonging to the late ripening cultivar, can be stored for up to seven months under the cold storage condition, while most of the early ripening apples can be stored for about one and a half months under cold storage [5].

Occurrence of definite organic acids determine the organoleptic properties and overall acceptability of fruits either by promoting or reducing the fruit taste [6]. Therefore, acidity has been considered as an important factor of fruit harvesting due to its direct influence on fruit quality particularly where acidity is crucial for further processing. Although fruit flavor largely depends on a multifaceted interaction of several metabolites, the ratio of sugars and acids are considered as a key determinant factor of fruit taste and sensory evaluation [7,8]. When at the expected level, this parameter will lead to repeated purchases and consumer loyalty [9]. In apple fruit, the primary soluble sugars are glucose, fructose, and sucrose, and the predominant organic acid is malate [10]. The early ripening apples are usually of high acidity and low sugar contents, which results in a low ratio of total sugars to total organic acids and reduce the market preference for fresh consumption. Thus, improvement of fruit taste for better consumption of early ripening apple is needed. Storage temperature is one of the most important factors affecting fruit storability [11] by regulating the rate of associated physiological and biochemical process. Many studies elucidated a prominent influence on the variation of organic acids and sugars in post-harvest fruit quality and shelf life [12–14].

Ultraviolet-C treatment (at hormetic dose) is a non-thermal, non-ionizing and safest practice inducing beneficial responses in fruits and vegetables and considered as an effective measure for maintaining postharvest quality [15]. Low-temperature storage and controlled atmosphere storage of apple fruit are common practices in recent years. However, UV-C treatment on apple has been rarely practiced, although it has promising effects on the extension of storage life of fresh horticultural crops and vegetables [16]. Previous studies stated the effectiveness of UV-C treatment on postharvest produce mainly by delaying ripening and senescence, maintaining relatively higher fruit firmness, flavonoid biosynthesis (e.g., anthocyanin) and increasing antioxidant ability and defense molecules [15,17]. Exposure to different doses of UV-C irradiations has reduced the titratable acidity of kiwifruits during cold-storage [18]. Furthermore, UV-C treatment lowered the amount of reducing sugar accumulation during low-temperature storage in potato tubers [19]. The accumulation and degradation of particular organic acid in mesocarp cell are modulated by both genetic and environmental factor. Several transcriptomics [20–22], metabolomics [22,23], proteomics [23,24], and quantitative trait loci (QTLs) [25,26] studies have been conducted recent years which described some of the mechanisms that control fruit acidity at the cellular level. As stated above, UV-C treatment has eminent effects on several quality aspects in fresh produce, it must regulate the induction of primary metabolites and interactive compounds. However, the underlying regulation of UV-C treatment on the malate metabolism in early ripening apple fruit during postharvest storage is still indistinct.

Considering ‘Hongyu’ apple is one of the traditional Chinese variety with high acidity and also an early ripening cultivar with shorter ripening period, it could be a good model to study apple fruit acidity modulation during post-harvest storage and find out the possible alternative treatment and storage condition which promote the fruit taste by regulating the fruit acidity of early ripening apple.

## Materials and methods

### Fruit materials and treatments

The ‘Hongyu’ apple fruits (*Malus domestica* Borkh.) were randomly harvested from different trees and different plant rows at commercial maturity stage (120 Dayes After Full Bloom) from an orchard located in Changping district, Beijing, P.R. China. The maturity stage was considered as the predefined date by the appropriate orchard management. The growing conditions of apple trees and the management practices during fruits development and ripening were controlled and maintained by the corresponding orchard management. Fruits of uniform size, appearance, and without any physical injuries or infections were selected and randomly divided into three groups (at least 15 fruits in each group). The fruits of different groups were subjected to the following postharvest treatments: (1) Cold storage: the fruits were stored at 4°C, 85–95% relative humidity (RH) throughout. (2) Room temperature storage: the fruits were stored at 20°C, 85–95% RH throughout. (3) UV-C treatment (storage at 20°C after treatment): A low-pressure mercury (LPM) UV light reactor was assembled using a UV tube (Ruisente Ltd., Tianjin, China); it delivered UV photons at 254 nm. The UV-C dose rate was determined by a digital ILT1700 radiometer (10 Technology Drive, Peabody, MA, USA). The different UV-C illumination doses were obtained by the exposure at a fixed distance. The apples were placed 20 cm below the lamps, and irradiated for 9.0 kJ m^-2^, and stored at 20°C after treatment, with a successive relative humidity of 85–95% throughout the storage period. The fruits were sampled at 14 d of storage. Fifteen fruits were sampled for individual treatment which has been divided into three biological replicates. Fruits were transported shortly to the laboratory, flesh sample was taken and immediately frozen by using liquid nitrogen and stored at -80°C until futher analysis.

### Organic acids and sugars measurement

The contents of organic acids and soluble carbohydrates were measured by following a modified method previously described by Lin et al. [19]. Briefly, a total of 1 g sample powder which was obtained by using high throughput tissue lapping apparatus (QM100, Wuzhou Ding Chuang technology, Beijing, China) homogenized with 4 mL of 80% ethanol. The mixture was extracted with ultra-sonication for 30 min at room temperature and centrifuged at 10,000 g for 15 min. Aliquots of 1 mL of the upper phase were dried under pure nitrogen. The residue was dissolved in 1 mL ddH_2_O and filtered through a membrane with 0.22 μm pore size. For sugar analysis, a volume of 10 μL for each sample was injected into the ion chromatograph (ICS-3000, Dionex, Sunnyvale, CA, USA) fitted with Carbo PacTMPA20 column (3 mm × 150 mm). The column temperature was 35°C, and the flow rate was 0.5 mL min^-1^. The gradient elution buffer was used as follows: A, ddH_2_O; B, 250 mmol L^-1^ NaOH; equal gradient of 92.5% A and 7.5% B were used for elution. A pulsed amperometric detector with gold electrode was used. For organic acid analysis, a volume of 25 μL for each sample was injected into the ion chromatograph (ICS-3000, Dionex, Sunnyvale, CA, USA) fitted with IonPac AS11-HC column (4 mm × 250 mm) (Dionex, Sunnyvale, CA, USA). The column temperature was 30°C, and the flow rate was 1 mL min^-1^. The gradient elution procedure was 0.8 mmol L^-1^ KOH, 0–12 min; 0.8–34 mmol L^-1^ KOH, 12–40 min; 34 mmol L^-1^ KOH, 40–50 min. The electrical conductivity detector was used for detection. Identification of the substances was according to the retention time (RT) of standard compounds. The contents of glucose, fructose, sucrose, malate, and oxalate were calculated by comparison with standard curves.

### Total RNA extraction

Total RNA was extracted using a modified CTAB method [27]. Quality of RNA was assesed by using gel electrophoresis. Purity of RNA detected by Nanodrop (OD_260/280_ ratio) spectrophotometer (IMPLEN, CA, USA). Qubit 2.0 Flurometer (Life Technologies, CA, USA) was used for accurate quantification of RNA concentration. RNA integrity was assessed by Agilent 2100 (Agilent Technologies, CA, USA).

### Library construction and RNA-Seq analysis

The RNA-Seq analysis was performed by following previously used methods described elaborately by onik et al. [28]. Total RNA from 4°C, 20°C post-harvest storage and UV-C treated fruits were pooled before library preparation and an equimolar quantity of total RNA from each sample was considered as one pool. Prior to cDNA library construction, poly-T oligo-attached magnetic beads were used to purify the mRNA, which was added to a fragmentation buffer to break mRNA into short segments of approximately 150–200 bp followed by cDNA synthesis using random hexamers and reverse transcriptase. Then buffer, dNTPs, RNase H and DNA polymerase I were added to synthesize the second strand cDNA. Both ends of sequencing adapters were ligated after terminal repairing of double-stranded cDNA fragments. The AMPure XP system (Beckman Coulter, Beverly, MA, USA) was used to purify the final cDNA library and selectively enriched by PCR enrichment and library quality was assessed on the Agilent Bioanalyzer 2100 system. The library preparations were sequenced by Novogene Bioinformatics Technology Co., Ltd. (Beijing, China) on an Illumina HiSeq PE150 platform. Three replications were used in each group for the precision of results. To obtain high-quality clean reads, raw reads were further filtered by removing adapter sequences, poly-N reads, and low-quality sequencesand all the clean reads were mapped to the Apple reference genome (https://www.rosaceae.org/organism/Malus/x-domestica v1.0) using TopHat [29]. Differentially expressed genes (DEGs) among the treatment groups werw identified using previously reported method [30], and *P*-values were adjusted to eliminate the false discovery rate (FDR) via multiple testing [31]. DESeq package (v1.10.1) was used and a corrected P-value threshold of 0.05 (using the Benjamini and Hochberg method) and |log2foldchang|>1 were considered for filtering the DEGs value. For gene expression quantification, the read numbers mapped to each gene were counted using HTSeq v0.6.1 and then normalized to FPKM (Fragments Per Kilobase of transcript sequence per Millions base pairs sequenced) [30]. For gene ontology (GO) enrichment analysis of differentially expressed genes, GOseq (v2.12) was used [32]. Kyoto Encyclopedia of Genes and Genomes (KEGG) enrichment analysis was performed using the KOBAS (v 2.0) software (Center for Bioinformatics, Peking University) [33].

### Quantitative real-time PCR (qRT-PCR)

First strand cDNA was synthesized from 1 μg RNA, after digestion of genomic DNA, using Bio-Rad iScript cDNA Synthesis Kit. qRT-PCR was performed using Power SYBR Green PCR Master Mix kit (Applied Biosystems, Foster City, CA, USA) on an ABI 7500 instrument (Applied Biosystems, Thermo Fisher Scientific, Waltham, MA, USA). The PCR reactetion protocol initiated by 5 min at 95°C, then followed by 45 cycles of 95°C for 10 s, 60°C for 10 s and 72°C for 15 s, and completed with a melting curve analysis program. No-template controls and melting curve analyses were included in every reaction. The *β*-*Actin* was used as an internal control. The comparative CT method (2^-ΔΔCT^ method) was used to analyze the relative expression levels of the different genes [34]. Primers used in this analysis were listed in S1 Table.

### Statistical analysis

Figures were drawn using Origin 8.6 (Microcal Software Inc., Northampton, MA, USA). The least significant difference (LSD) at 0.05 level was calculated by DPS 7.05 (Zhejiang University, Hangzhou, China).

## Results

### Variation of organic acids and sugars contents in response to temperature and UV-C treatment in apple

**Fig 1:**
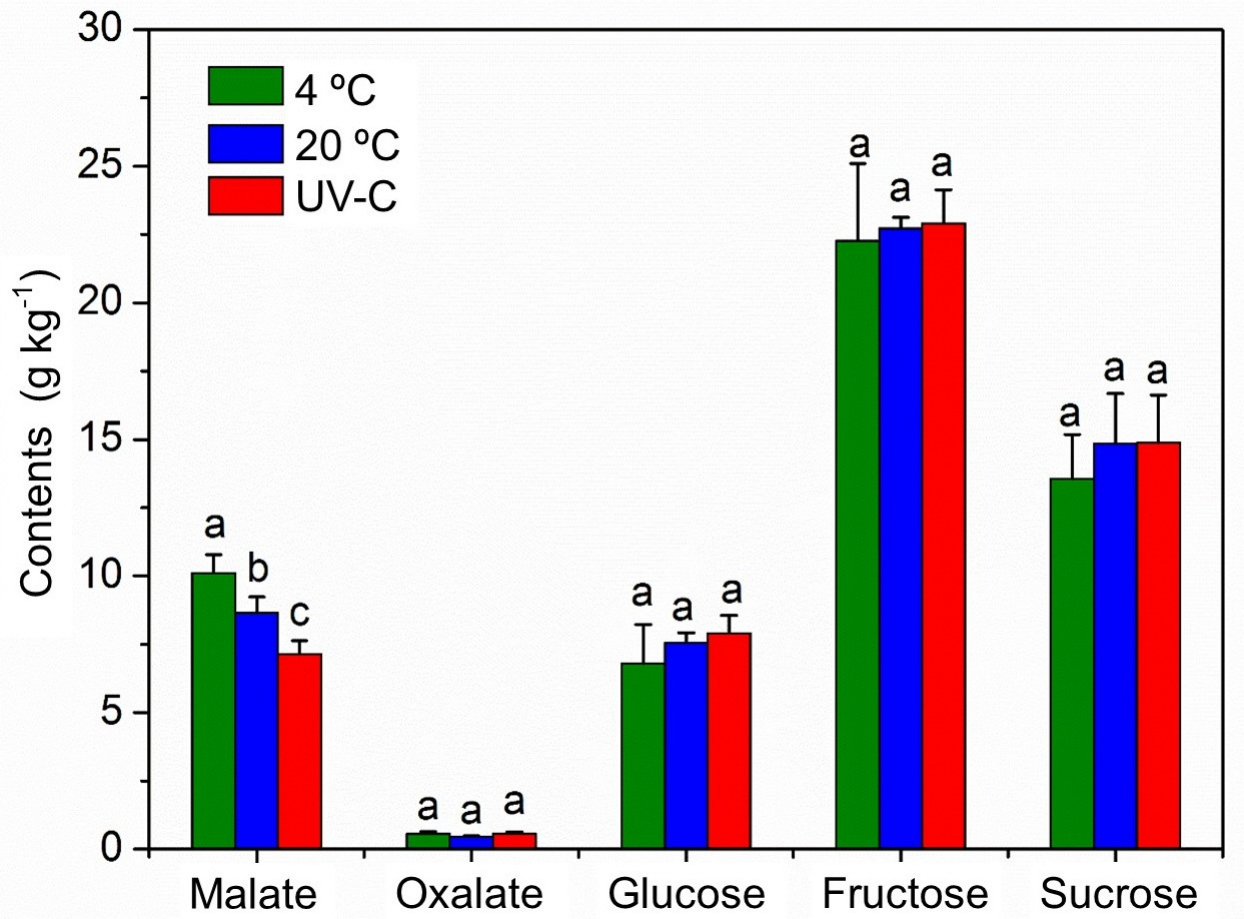
Variation of sugars (glucose, fructose and sucrose) and organic acids (oxalate and malate) in apple fruit under different temperature storage and postharvest UV-C treatment. The error bar represents the standard error of the mean value of three biological replicates. Same letters indicated the mean values are not statistically different according to the least significant difference test (p < 0.05).

Among the primary organic acids and sugars present in apple fruit, malate, oxalate, glucose, sucrose and fructose content in different treatment group (4°C, 20°C, and UV-C treatment following 20°C storage after treatment) were measured. Results indicated that malate and fructose was the most prominent in content. The oxalate contents were below 0.5 g kg^-1^, while the malate contents varied among 7 to 12 g kg^-1^. Compared to the fruits stored at 4°C, the malate content in fruits stored at 20°C was significantly decreased at 14 d of storage. According to the results, the content of glucose, fructose and sucrose were not significantly varied among the different treatments. However, glucose and sucrose were slightly higher in 20°C stored fruit and UV-C treated fruit comparing 4°C temperature stored fruit. Considering the significant variation of malic acid content, all the samples of different temperature storage and UV-C treated fruits at 14 d of storage was considered for RNA-seq analysis. The variation of acids and sugars content was shown in Fig 1.

### RNA-Seq libraries and the differently expressed genes (DEGs) analysis

Three replications of each treatment were used for RNA-Seq analysis to investigate the molecular mechanism underlying malate metabolism in apple fruit during postharvest storage. After removing adapter reads, low-quality regions, and possible contamination, more than six clean gigabases with a GC percentage above 46.84% and a Q20 percentage above 95.81% were obtained. The proportion of clean reads in the apple transcriptome libraries that mapped to the apple reference genome ranged from 87.87% to 93.39%, while the adjusted proportion ranged from 73.35% to 78.85% (Table 1).

**Table 1:** Quality evaluation of RNA-Seq data.

Groups	Replicates	Clean bases (G)	Error rate (%)	Q20 (%)	GC content (%)	Total mapped (%)	Uniquely mapped (%)
**4°C**	A	7.80	0.02	97.42	46.84	94.31	78.85
	B	7.33	0.02	96.5	47.1	93.39	77.65
	C	9.19	0.02	96.55	47.12	93.29	77.51
**20°C**	A	8.47	0.02	95.81	48.20	90.05	75.19
	B	7.31	0.02	95.43	47.74	87.87	73.35
	C	7.37	0.02	97.30	47.20	92.36	76.89
**UV-C**	A	7.79	0.02	97.19	47.63	92.91	77.48
	B	7.42	0.02	96.53	47.91	90.92	75.53
	C	6.55	0.02	96.87	47.85	89.39	74.39

**Fig 2:**
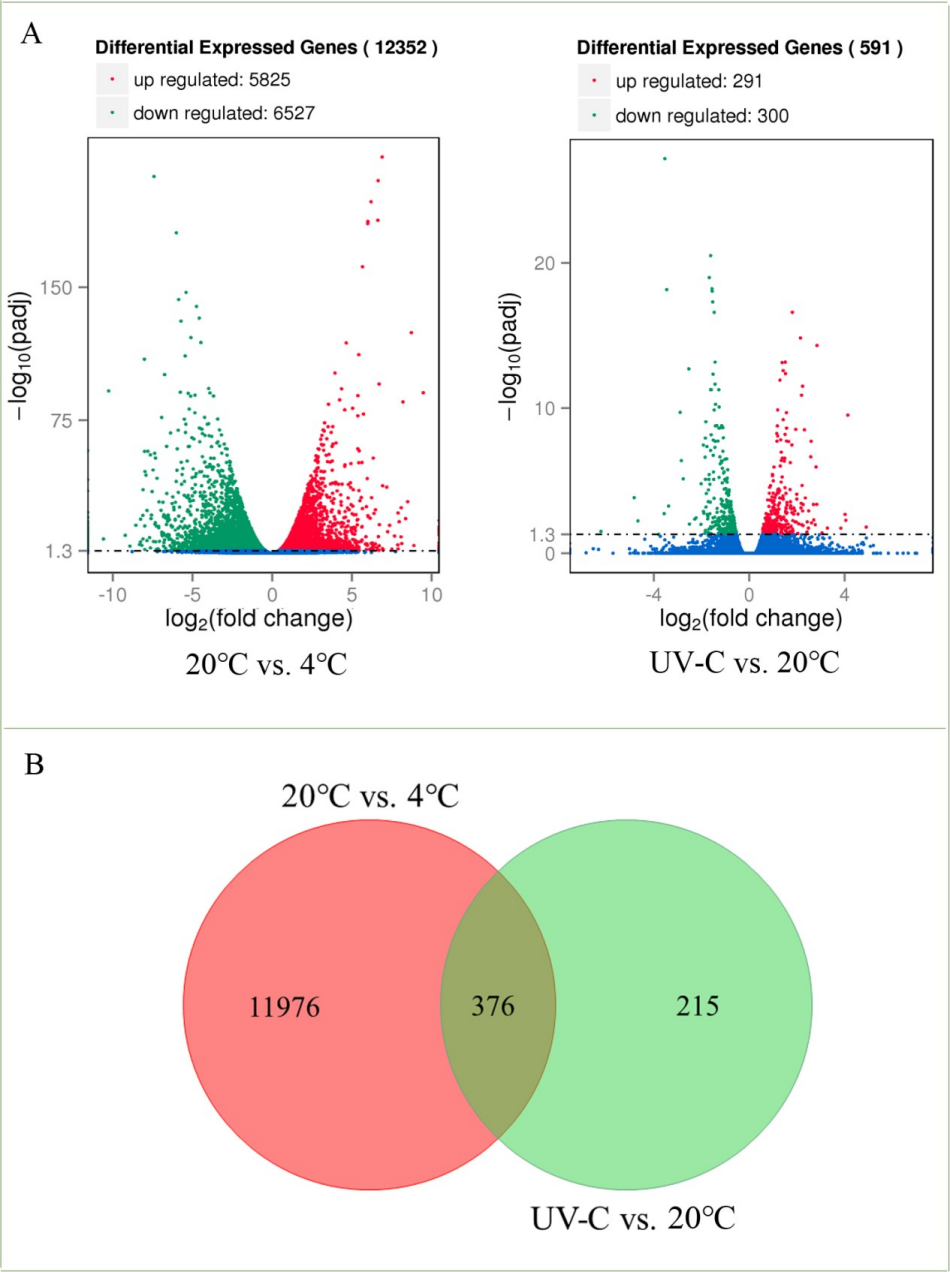
Volcano plots and venn diagrams of DEGs. DEGs of 20°C vs. 4°C and UV-C vs. 20°C displayed by volcano plots (A). The horizontal and vertical coordinates indicate the fold change differences and adjusted *p*-values; respectively for differential expression of genes. The up-regulated genes are represented by red dots, and the down-regulated genes are represented by green dots. Genes without significant differences are indicated by blue dots. Venn diagrams showing the overlap in DEGs between 20°C vs. 4°C and UV-C vs. 20°C groups (B).

A total of 70,163 unique genes had perfect matches with apple genes, including 6,648 new predicted genes (S2 Table). We compared the transcript levels of each gene among different samples, 12,352 and 591 DEGs were found in the clusters of 20°C vs. 4°C and UV-C vs. 20°C, respectively (Fig 2A). 5,825 up-regulated and 6,527 down-regulated DEGs were observed in 20°C vs. 4°C cluster, while 291 up-regulated and 300 down-regulated DEGs were found in UV-C vs. 20°C cluster. Venn diagram showed that 376 DEGs were overlapped between clusters of 20°C vs. 4 ºC and UV-C vs. 20°C (Fig 2B). 11,976 and 215 DEGs were exclusively shown in 20°C vs. 4°C cluster and UV-C vs. 20°C cluster, respectively.

### GO classification of the DEGs in apple during postharvest storage

**Fig 3:**
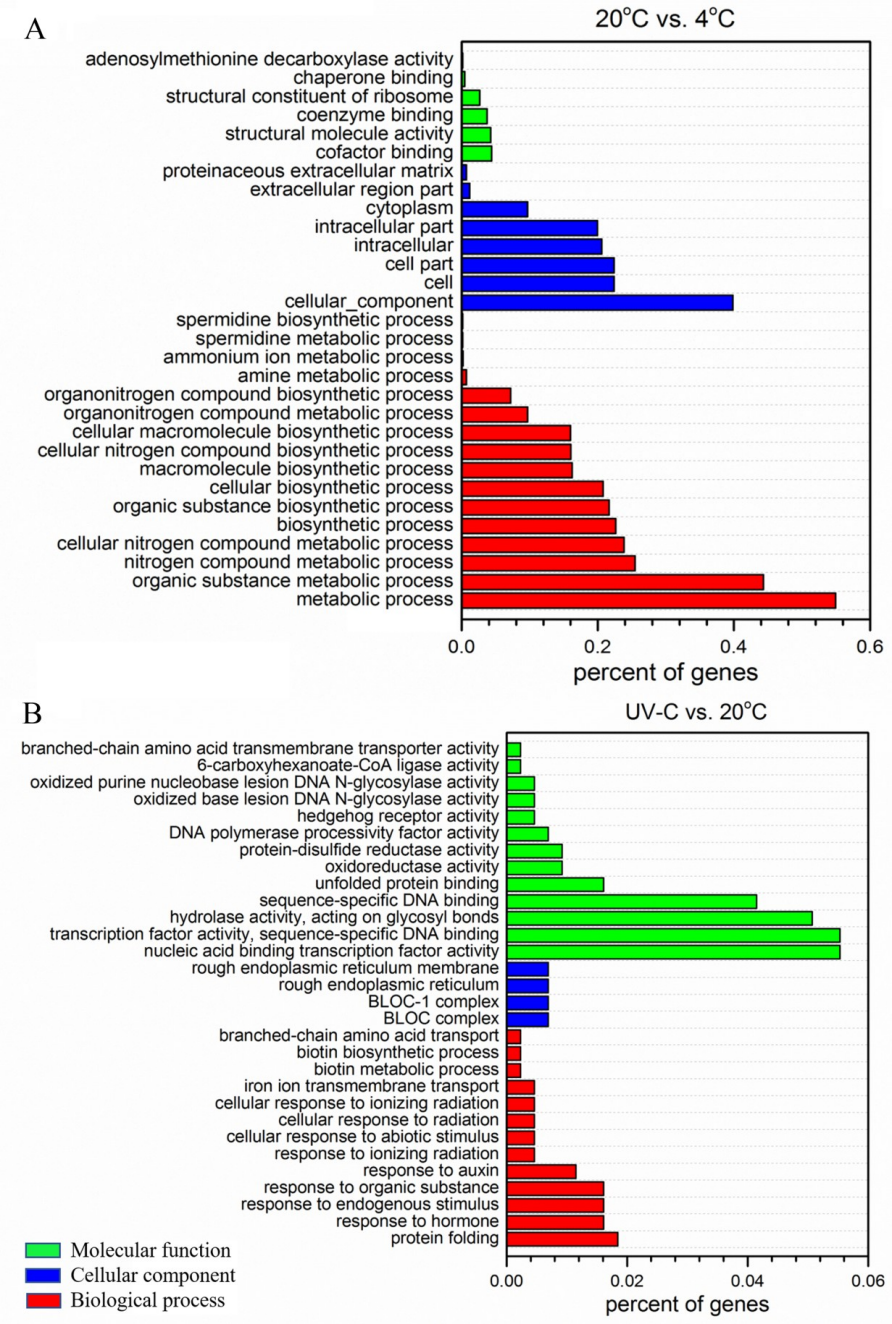
**Classification of GO terms concerning differently expressed genes in apple under different temperature (A) and UV-C treatment (B) during postharvest storage.** The GO terms were classified into three categories, including molecular function, cellular component, and biological process. Top 30 enriched GO terms were exhibited in each cluster.

We further assigned gene ontology (GO) terms to annotated DEGs in apple during postharvest storage (S3 Table). In 20°C vs. 4°C cluster, the largest terms within the molecular function category were ‘cofactor binding’, ‘structural molecule activity’, and ‘coenzyme binding’. Within the cellular component category, ‘cell’, ‘cell part’ and ‘intracellular part’ were the three most abundant terms. Within the biological process category, the most highly represented terms were ‘metabolic process’, ‘organic substance metabolic process’, and ‘nitrogen compound metabolic process’ (Fig 3A). In UV-C vs. 20°C cluster, the largest terms within the molecular function category were ‘nucleic acid binding transcription factor activity’, ‘transcription factor activity, sequence-specific DNA binding’, and ‘hydrolase activity, acting on glycosyl bonds’. Within the cellular component category, ‘rough endoplasmic reticulum membrane’ and ‘BLOC-1 complex’ were the two most abundant terms. Within the biological process category, the most highly represented terms were ‘protein folding’, ‘response to hormone’, ‘response to endogenous stimulus’, and ‘response to organic substance’ (Fig 3B).

### KEGG pathway enrichment of the DEGs in apple during postharvest storage

**Fig 4:**
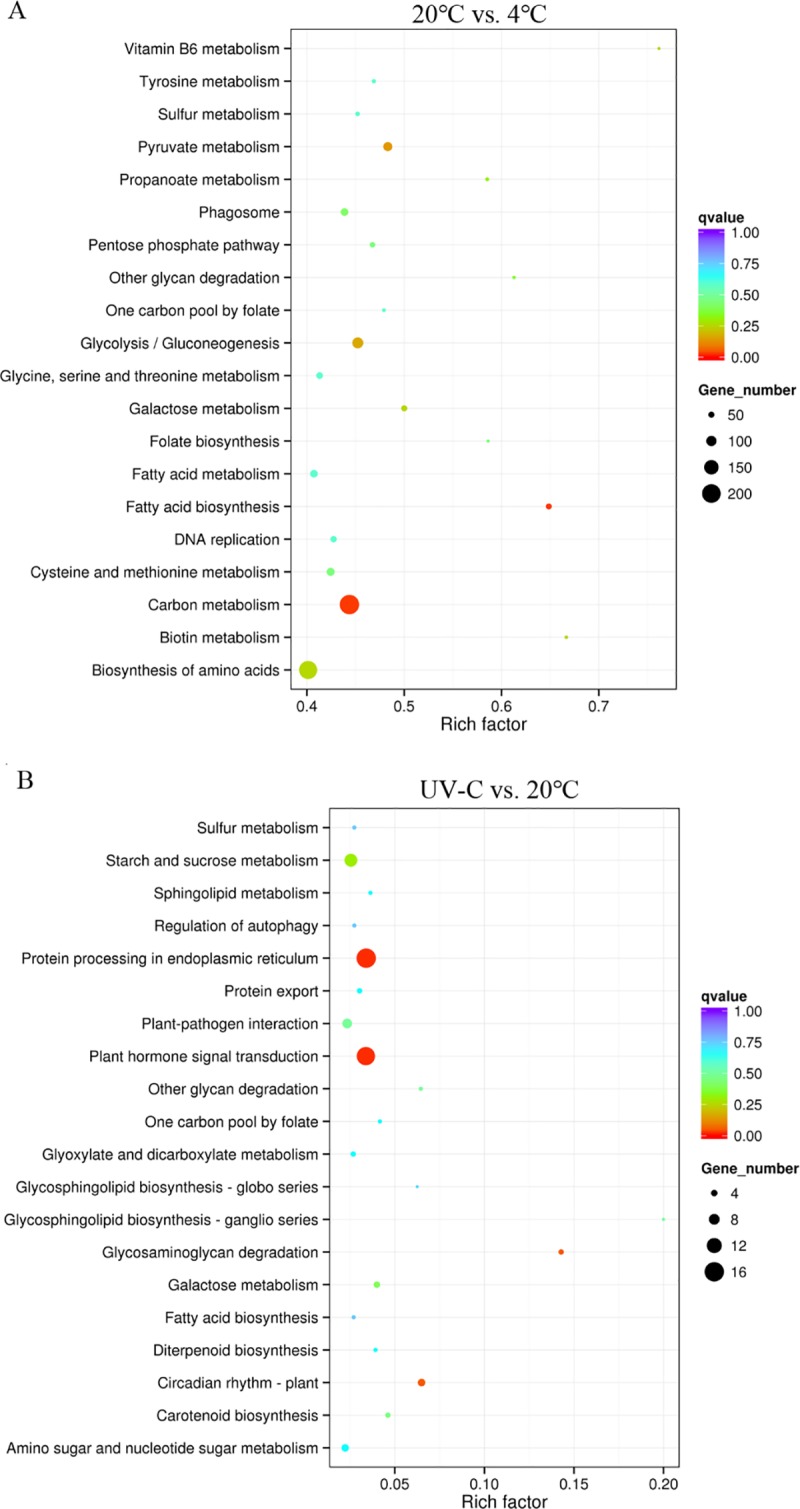
**KEGG pathway enrichment concerning differently expressed genes in apple under different temperature (A) and UV-C treatment (B) during postharvest storage.** Top 20 enriched KEGG pathways were exhibited in each cluster. Rich factor is the ratio of DEGs counts to this pathway in the annotated genes counts. The more the q-value is close to zero, the more significant is the enrichment.

To identify the biological pathways activated in selected apple fruits, we mapped the annotated sequences to the reference pathways in the KEGG database (S4 Table) [35]. The top 20 most enriched pathways between the two groups were shown in Fig 4. In 20°C vs. 4°C cluster, the ‘carbon metabolism’, ‘fatty acid biosynthesis’, ‘pyruvate metabolism’, ‘glycolysis’ and ‘biosynthesis of amino acids’ were the five most enriched pathways (Fig 4A). In UV-C vs. 20°C cluster, the ‘plant hormone signal transduction’, ‘protein processing in endoplasmic reticulum’, ‘Glycosaminoglycan degradation’, ‘Circadian rhythm’ and ‘starch and sucrose metabolism’ were the five most enriched pathways (Fig 4B).

### Malate metabolism in response to temperature and UV-C treatment in apple during postharvest storage

According to our results, the malate content was significantly influenced by cold-storage and UV-C treatment. Compared to the fruits stored at room temperature, the malate content was significantly higher in the fruits stored at cold-storage. The expression of tricarboxylic acid cycle (TCA) related genes, including *citrate synthase* (*CS*), *aconitase* (*ACO*), *isocitrate dehydrogenase* (*IDH*), *succinate dehydrogenase* (*SDH*), and the malate synthesis-related genes, including *phosphoenolpyruvate carboxylase* (*PEPC*) and *NAD-malate dehydrogenase* (*NAD-MDH*) were up-regulated, whereas a significant down-regulation was showed by malate degradation related genes, *NADP-malic enzyme* (*NADP-ME*) and *phosphoenolpyruvate carboxylase kinase* (*PEPCK*). On the other hand, a significant declination of malate content in UV-C treated fruits was observed in contrast to the fruits stored at room temperature. Consequently, no significant difference was observed in the expression of TCA cycle and malate synthesis-related genes. The expression of *NADP-ME* and *PEPCK* were significantly induced by UV-C treatment, which might be the key genes regulating malate degradation in UV-C treated apples during postharvest storage (Table 2).

**Table 2:** Differently expressed genes related to malate metabolism in apple under different temperature and UV-C treatment during postharvest storage.

Enzymes	Gene ID	FPKM ratio (4°C/20°C)	FPKM ratio (UV-C/20°C)
**NADP-malic enzyme (NADP-ME)**	MDP0000258977	0.52	1.31
	MDP0000291902	0.40	0.75
	MDP0000376988	0.45	1.42
	MDP0000568928	0.45	2.33
	MDP0000132833	0.66	1.01
	MDP0000168801	0.43	0.80
**NAD-malate dehydrogenase (NAD-MDH)**	MDP0000143956	2.46	1.75
	MDP0000170418	2.29	1.11
	MDP0000174740	1.62	0.89
	MDP0000195734	1.97	0.77
	MDP0000197620	1.86	0.93
	MDP0000278198	5.56	0.76
	MDP0000532379	3.54	1.48
	MDP0000568449	2.85	1.24
**Phosphoenolpyruvate carboxylase (PEPC)**	MDP0000073810	5.16	0.74
	MDP0000256162	0.47	0.80
	MDP0000270146	0.34	0.81
	MDP0000291654	2.56	1.60
	MDP0000305440	5.65	0.72
	MDP0000748981	0.71	0.80
**Phosphoenolpyruvate carboxylase kinase (PEPCK)**	MDP0000122593	0.08	0.70
	MDP0000661864	0.16	2.44
**NAD-malic enzyme (NAD-ME)**	MDP0000231868	2.13	1.45
	MDP0000453139	2.02	1.04
**Pyruvate dehydrogenase (PDH)**	MDP0000139720	2.12	0.90
	MDP0000192364	0.56	0.98
	MDP0000409645	0.46	0.65
**Citrate synthase (CS)**	MDP0000321473	2.45	0.98
**Aconitase (ACO)**	MDP0000255452	2.17	0.83
	MDP0000279281	2.87	0.69
**Isocitrate dehydrogenase (IDH)**	MDP0000169990	5.79	1.29
	MDP0000184745	4.52	1.25
	MDP0000295370	0.59	0.97
	MDP0000600681	5.08	1.25
	MDP0000389969	2.65	0.98
**Succinate dehydrogenase (SDH)**	MDP0000188391	2.54	1.05
	MDP0000439248	0.44	0.86
	MDP0000612264	0.43	0.80

**Fig 5:**
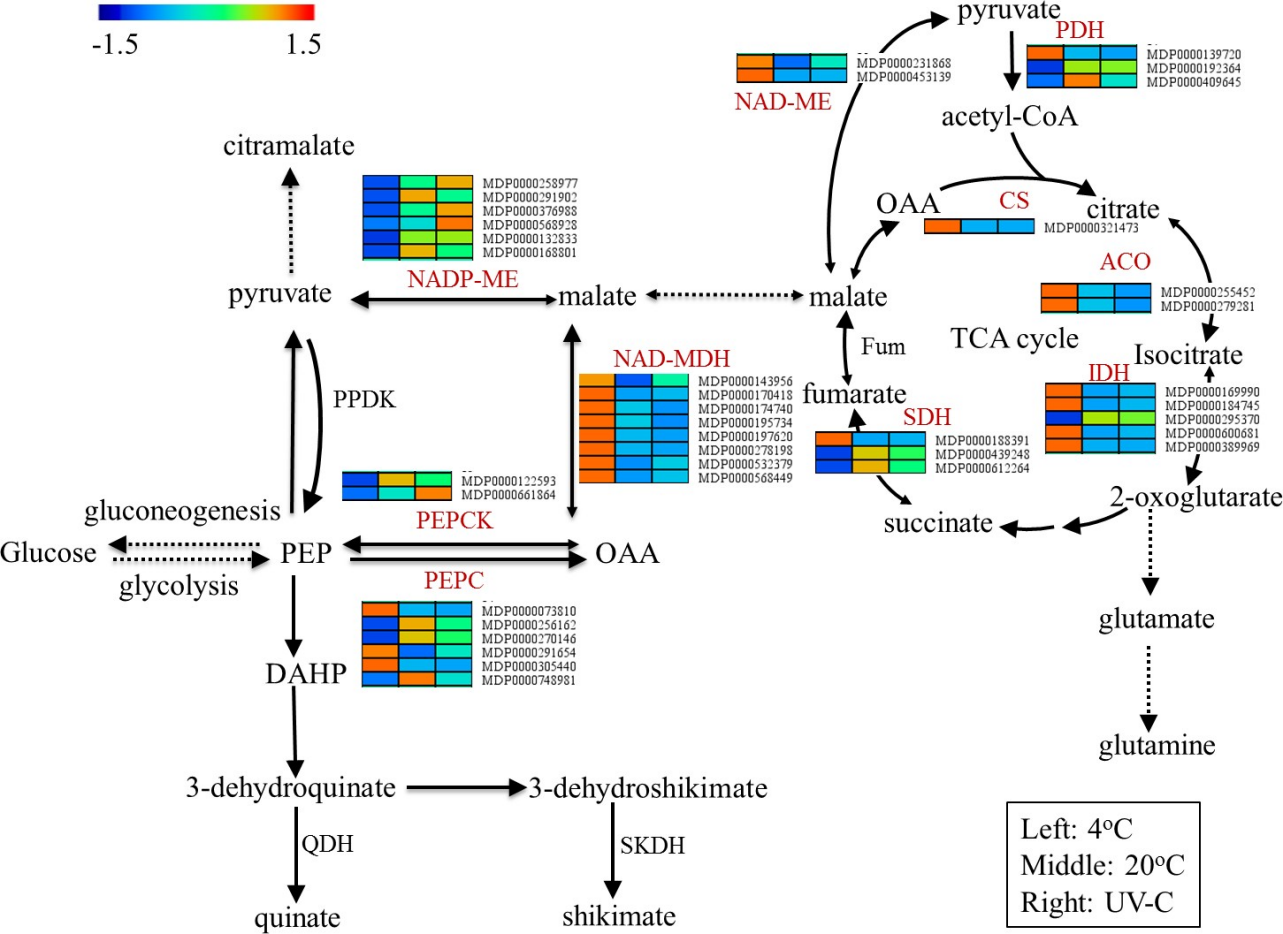
The regulation of malate metabolism pathway in apple under different temperature and UV-C treatments during postharvest storage. The rectangles from left to right represent 4°C, 20°C and UV-C treated fruits. ACO, Aconitase; CS, Citrate synthesis; IDH, Isocitrate dehydrogenase; PEPC, Phosphoenolpyruvate carboxylase; PEPCK, Phosphoenolpyruvate carboxylase kinase; PDH, Pyruvate dehydrogenase; NAD-MDH, NAD-malate dehydrogenase; NADP-ME, NADP- malic enzyme; PPDK, Pyruvate orthophosphate dikinase; SDH, Succinate dehydrogenase; SKDH, Shikimate dehydrogenase.

Briefly, higher expression of TCA cycle and malate synthesis-related genes in the cold stored fruit (4°C) resulting in promotion of malate synthesis. On the other hand, higher degradation of malate was taken place by up-regulation of *NADP-ME* and *PEPCK* in UV-C treated fruits. The interactive expressions of the genes in primary pathway of malate metabolism regarding different storage temperature and postharvest UV-C treatment were shown in Fig 5.

### Quantitative Real-Time PCR (qRT-PCR) validation of DEGs

**Fig 6:**
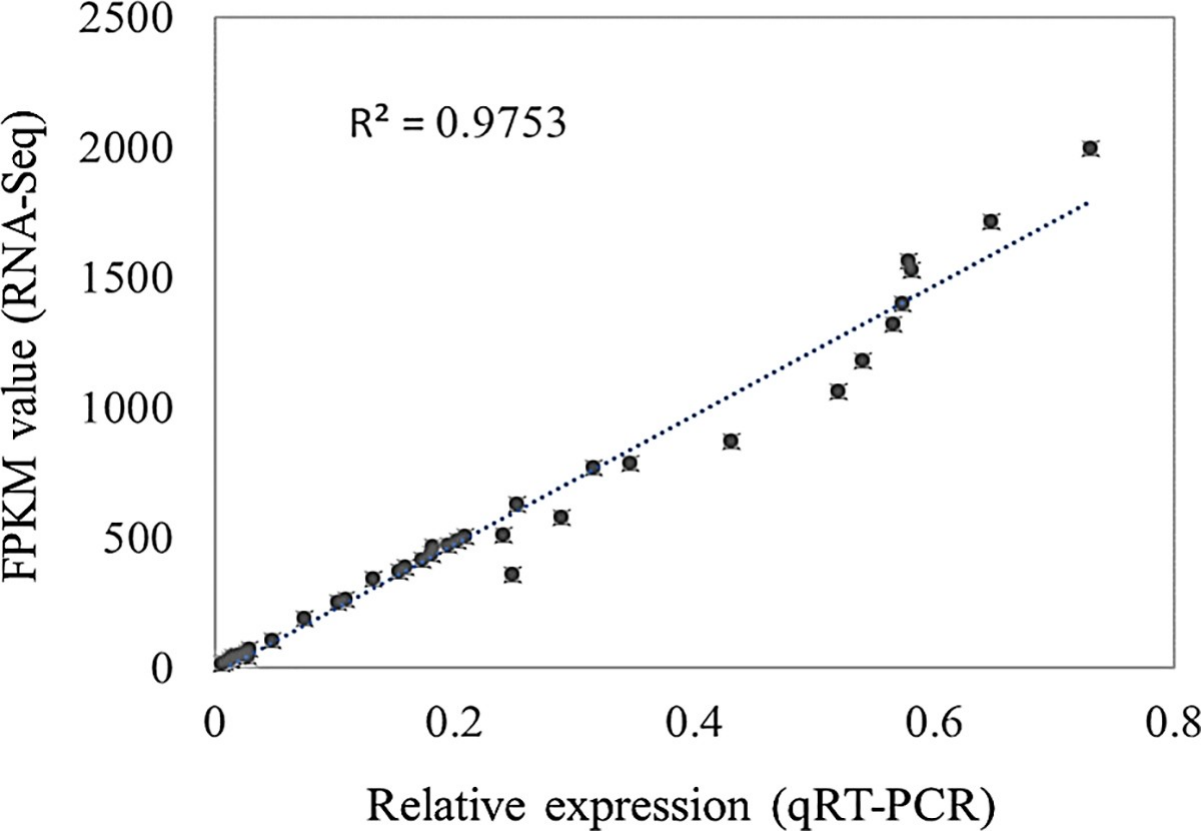
Correlation analysis of relative expression levels determined by RNA-seq and qPCR on selected genes *NADP-ME* (MDP0000568928), *NAD-MDH* (MDP0000174740), *PEPCK* (MDP0000661864) and *PEPC* (MDP0000073810). All qPCR reactions were carried out by using three independent biological replication and repeated three times. Relative expression levels were calculated from CT values according to the 2^-ΔΔCT^ method. Actin was used as the reference gene for these qPCR experiments.

To validate the credibility of the gene expression profiles obtained from RNA-seq analysis, we selected the DEGs involved in the malate metabolism pathway for qRT-PCR using specific primers to confirm the gene expression changes detected in the transcriptome analysis. The exact fold change of DEGs at several data points varied between RNA-seq and qPCR methods. However, the overall expression trends were strongly consistent (Pearson correlation coefficients R^2^ = 0.9753) (Fig 6), confirming the reliability of the RNA-seq results.

## Discussion

Considering the health benefits associated with apple progressively understood through consumers, demand is increasing for cultivars with preferred quality features, such as size, texture, flavor and nutritional value [36]. For an apple cultivar, fruit quality characteristics are underlain by metabolite composition and contents at maturity and during postharvest storage. The content of predominant organic acid and metabolism also plays an important role in food processing [37] and quality [38]. The early ripening apples are mostly used for fresh marketing because of short storage life, but they are usually with high acidity and low sugar contents [5,39].

UV-C treatment has already been established as a positive regulator for postharvest preservation of several fruits and vegetables, such as tomato [40], strawberry [41], apple [42], mango [43] and peach [44] by inducing plant defense response, inhibition of particular microbes as bacteria, disease resistance gene regulation and modulating antioxidant capacity. It has been reported that, exposure to short wavelength UV-C (200–280 nm) irradiation at low (hormetic) doses, resulted in the enhancement of antioxidant capacity, anthocyanin content, and phenolic content in mangoes, edible mushrooms, strawberries, blueberries and grape [45]. UV-C irradiation in the range of 0.125–9 kJ m^−2^ showed beneficial effects including increased resistance to pathogens, delayed ripening and senescence, and increased the content of beneficial phytochemicals [15,46]. In general, our results showed significant differences between the UV-C treated fruits over low temperature stored fruits and room temperature stored fruits but the sugar contents were not significantly diverged between different storage conditions. The similar trend was observed in a previous study on the action of UV-C on ‘Trust’ tomato fruit at mature-green stage where the fruit acidity (pH and titratable acidity) was apparently more sensitive to the treatment, with a significantly lower acidity in UV-C treated fruit [47]. However, another study showed higher malic acid in UV-C treated tomato fruits at breaker stage which was reduced in UV-treated vine-ripe tomato after 15d of storage [48]. In addition, titratable acid content was comparatively higher in UV-C treated ‘Fuji’ apple than those of control fruit stored at 20°C [49]. Following different effects observed in different horticultural produce, important things must be taken into consideration to achieve successful implementation such as plant parts used for treatment, dose, cultivar specificity, developmental stages, harvesting date, pre-harvest factors and postharvest storage conditions [49].

The final malate content of fruit is generally determined by the net balance of acid synthesis, degradation, and compartmentation [50,51]. Malate biosynthesis occurs primarily in the cytosol and is catalyzed by PEPC and NAD-MDH [52]. Loss of acidity signifies decarboxylation associated with carboxylates which can occur with the conversion of tricarboxylates into dicarboxylates, but additionally through decarboxylation of the malate and oxaloacetic acid (OAA), leading to the actual degradation involving organic acids. Decarboxylation connected with OAA as well as malate enables the production of phosphoenolpyruvic acid (PEP), which may additionally originate from the activity with PEPCK, directed to the gluconeogenesis process [6]. Those findings supported by the study indicating the involvement of PEPCK in the dissimilation of malate of several fleshy fruits [24]. The results also revealed a potential association between PEPCK enzyme activity, and malate loss in raspberry, blueberry, and red currant fruits. Involvement of PEPCK was observed in the dissimilation of malate and possibly in the lack of malate in low acid apple cultivars [53]. Another study demonstrated continuous revenues of PEPCK in maize protoplasts through the ubiquitin protease pathway, may act for rapid reversal of PEPC phosphorylation, thus considered as another level of control for malate metabolism [54]. The instigation of PEP can also be taken place by the conversion of pyruvate through pyruvate orthophosphate dikinase (PPDK) activity and the required pyruvate for PPDK may be supplied through the carboxylation of malate by a cytosolic NADP-dependent malic enzyme (NADP-cytME) [6]. Involvement of NADP-cytME in the decrease in malate content during the ripening has been demonstrated in several fruit species [6,55]. NADP-cytME appears to be involved in the lack of malate in the ripe pulp of low acid apple cultivars [56]. Increasing the activity of NADP-ME enzymes has also been observed during grape berry ripening, indicating the functions of this enzyme in malate degradation [57]. Thus, the rapid decrease in malate content during fruit ripening has usually been attributed to its degradation by cytosolic NADP-ME [55].

The *NADP-ME* gene promoter is activated by different effectors, such as fungal elicitors, UV irradiation and wounding, and agents producing redox perturbations in bean [58,59]. NADP-ME activity was also increased by UV-B radiation in bean [60] and in maize [61,62]. Our results showed that the increased expression of *NADP-ME* played a significant role in malate metabolism during postharvest storage in UV-C treatment of apples, which provides further evidence that *NADP-ME* has a role in response to UV-C stress. As a primary contributor in fruit flavor development, maintaining the sugars and acids contents is of greatest interest to select a postharvest treatment. In general, the postharvest condition or treatment that promotes TSS: TA would have a positive effect on flavor development. Therefore, the significantly higher sugar: acid ratios in the UV-C treated fruit of this cultivar under the described conditions, UV-C treatment positively affected fruit quality. Although there are some homology and as well as deviation of our results with previous studies, the following trend of malate degradation through the UV-C treatment during postharvest storage at room temperature and stimulated response of the genes involved in the degradation of malate in fruit would lead to a better fruit quality of particular cultivar. The potential effects of UV-C hormesis should therefore be considered as a way to enhance quality without negatively impacting quality attributes of early ripening apple fruit. It may also be possible that UV treatment stimulates a class of compounds that could counteract the positive sugar acid ratio attribute. However, for successful implementation of UV-C treatment on early harvested apple fruit, the deviation of the current study must be taken into consideration for further study by adjusting the dose, storage parameters and cultivars of apple fruit.

## Conclusions

Low temperature maintained significantly higher malate contents by up-regulating the expression of genes (*NAD-MDH* and *PEPC*) related to the TCA cycle and citrate synthesis and down-regulating the expression of *NADP-ME* and *PEPCK*. UV-C treatment significantly increased the ratio of total sugars to total acids compared with room temperature stored fruits, which was carried out by the up-regulation expressions of *NADP-ME* and *PEPCK*. This finding suggested UV-C as an effective measure to maintain and promote the fruit taste during post-harvest storage of early ripening apple cultivar at room temperature.

## Supporting information

S1 Table Primers used for qRT-PCR analysis. (PDF)

S2 Table List of annotated DEGs. (XLSX)

S3 Table List of GO enrichment analysis results. (XLSX)

S4 Table List of KEGG enrichment analysis results. (XLSX)
