# Supplementary material for: UV-C treatment promotes quality of early ripening apple fruit by regulating malate metabolizing genes during postharvest storage
Source: PLoS One. 2019 Apr 16;14(4):e0215472. doi: 10.1371/journal.pone.0215472 (PMC6467447; doi:10.1371/journal.pone.0215472)
Supplement: S1 Table — (PDF) [file pone.0215472.s001.pdf]

S1 Table. Primers used for qRT-PCR analysis.

Genes	Forward primer	Reversed primer
MdActin	TGACCGAATGAGCAAGGAAATTACT	TACTCAGCTTTGGCAATCCACATC
MdNAD-MDH	CTTAGATGCTCTTGGCCTTTCC	TCAGCTGCTTTTCTCACACATTG
MdNADP-ME	GTACAGCCCTTCCTATCGAAGTTT	TCTTGAACCCGGTACAACCAA
MdPEPCK	CTTCGAGGTGTTCATGAATCACA	TTGATCGGTTCACATGACATGG
MdPEPC	CGAGAGACGGGTTGTGTCC	TGCACAGCAATCTGATCACAG
